# Dual transcriptomic analysis unraveling the immune landscape and host-pathogen interactions during *Mycobacterium tuberculosis* infection

**DOI:** 10.1016/j.isci.2025.114102

**Published:** 2025-11-17

**Authors:** Chenyan Shi, Xiaoqian Liu, Dan Chen, Tong Wang, Yu Wang, Ningjian Cai, Zhaodong Li, Yunlong Hu, Yi Cai, Xinchun Chen

**Affiliations:** 1 School of Public Health, Shenzhen University Medical School, Shenzhen, China; 2 Guangdong Provincial Key Laboratory of Infection Immunity and Inflammation, Department of Pathogen Biology, Shenzhen University Medical School, Shenzhen, China; 3 College of Life Science and Technology, Wuhan Polytechnic University, Wuhan, Hubei, China; 4 Institute of Clinical Medicine, The Second Affiliated Hospital of Hainan Medical University, Haikou, Hainan, China

**Keywords:** Immunology, Microbiology, Transcriptomics

## Abstract

Elucidating the host-pathogen interactions is critical for uncovering the mechanisms controlling *Mycobacterium tuberculosis* (*Mtb*) infection. Using dual RNA-seq with fluorescent *Mtb*, we simultaneously profiled macrophage and bacterial transcriptomes to resolve dynamic intracellular responses. Macrophages containing dead *Mtb* exhibited strong immune activation, including enhanced antigen presentation and lysosomal function, whereas macrophages harboring live *Mtb* showed persistent NF-κB signaling and metabolic reprogramming. *Mtb* counteracted host defenses through upregulation of DNA repair genes and manipulation of extracellular matrix signaling via SPP1 and integrins, alongside tryptophan catabolism and lipid binding pathways supporting adaptation. Cross-species correlation analysis revealed coordinated transcriptional programs, notably a strong inverse association between *Mtb* aromatic compound catabolism and host receptor tyrosine kinase signaling. Additional correlations linked bacterial metabolism with host lipid transport and steroid biosynthesis. Together, these results provide a high resolution view of macrophage and *Mtb* transcriptional interplay defining bacterial persistence versus immune clearance.

## Highlights

• Dual RNA-seq maps coordinated transcriptional programs in macrophage-*Mtb* interplay
• Lysosomal activation and antigen presentation drive effective macrophage control
• Macrophages harboring live *Mtb* show persistent inflammation and metabolic shift
• Cross-species correlations couple host RTK signaling to *Mtb* aromatic catabolism

## Graphical abstract

**Figure:**
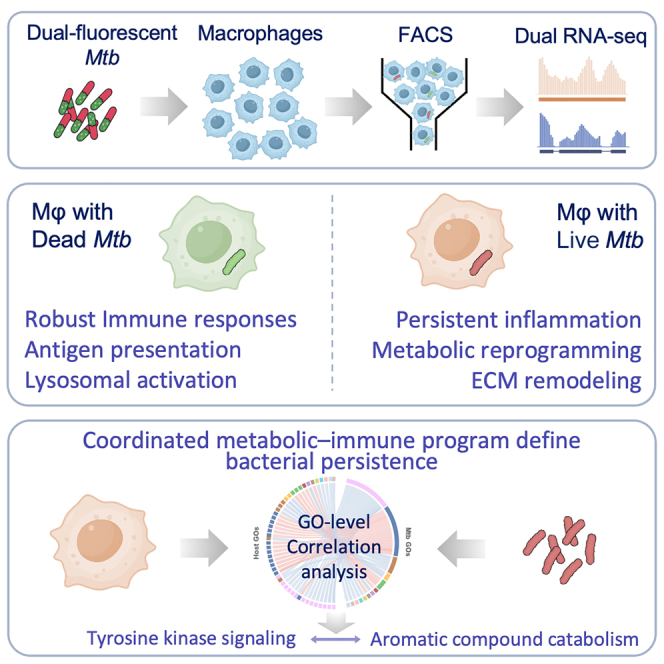

## Introduction

Tuberculosis (TB), caused by *Mycobacterium tuberculosis* (*Mtb*), remains one of the most formidable global health challenges, claiming millions of lives each year.^1^ Central to the pathogenesis of TB is the complex interplay between *Mtb* and the host immune system, particularly the role of macrophages. As the first line of defense, macrophages are tasked with detecting, engulfing, and destroying *Mtb* upon infection.^2^ However, the relationship between *Mtb* and macrophages is dual-faceted.^3^

Understanding the molecular basis of this host-pathogen tug-of-war remains a critical goal in TB research, with important implications for the development of host-directed therapies. Dual RNA-seq is a powerful tool that allows the simultaneous, high-precision, and in-depth profiling of both host and pathogen transcriptomes without the need for prior separation. This approach facilitates the examination of temporal shifts in gene expression and helps elucidate how pathogen manipulates host cellular processes to establish a successful infection.^4^ Previous applications of dual RNA-seq, such as the study by Pisu et al., revealed distinct transcriptional profiles between alveolar and interstitial macrophages isolated from the lungs of *Mtb*-infected mice, along with metabolic differences in their respective intracellular bacteria.^5^ However, the study lacked resolution to distinguish host responses by bacterial viability or infection stage due to the complexity of *in vivo* systems.

To address these gaps, we employed an *in vitro* infection model using THP-1 macrophages challenged with double-fluorescent-engineered H37Ra. This allowed us to distinguish host cells harboring live versus dead intracellular bacteria and perform dual RNA-seq at 48 and 72 h post-infection. Our study design integrates three complementary layers of analysis: (1) profiling the immune states of macrophages in response to live versus dead *Mtb*; (2) examining temporal changes in these responses over the course of infection; and (3) investigating co-regulated gene expression patterns between host and surviving intracellular bacteria. Through this multi-dimensional approach, we aim to uncover transcriptional correlates of bacterial persistence and host immunity, providing a refined framework for understanding TB pathogenesis at the host-pathogen interface.

## Results

**Figure 1:**
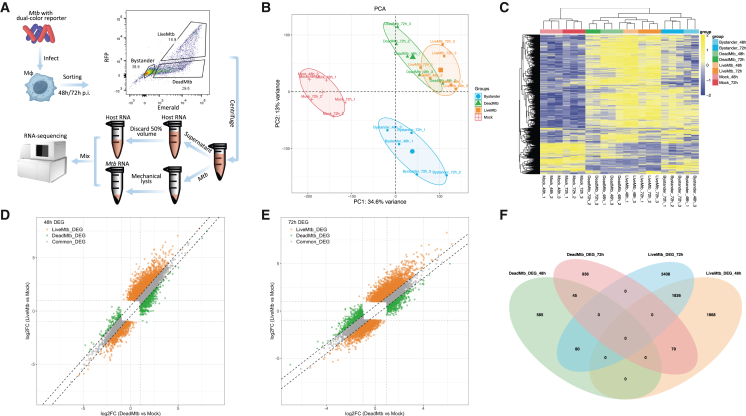
Overview of the transcriptomic profiles of PMA-differentiated THP-1 macrophages infected with H37Ra (A) Flowchart of study design. (B) PCA plot of gene expression profiles in Mock, Bystander, DeadMtb and LiveMtb group on at 48 and 72 h. (C) Heatmap of gene expression levels in the four groups at 48 and 72 h. (D) Dot plot of the DEGs based on the Log2FC between LiveMtb and Mock, as well as between DeadMtb and Mock at 48 h and (E) 72 h. (F) Venn diagram of the defined DEG of DeadMtb and LiveMtb at 48 and 72 h.

We performed dual RNA-seq on phorbol 12-myristate 13-acetate (PMA)-differentiated THP-1 macrophages sub-populations infected with double-fluorescent-engineered H37Ra *in vitro*. This strain constitutively expressed Emerald (green) and expressed TagRFP (red) only upon tetracycline induction, enabling discrimination of bacterial viability by fluorescence-activated cell sorting (FACS) (Figure 1A). At 48 and 72 h post-infection, THP-1-derived macrophages were sorted into three groups: uninfected cells (Bystanders; Emerald^−^/TagRFP^−^), cells harboring dead *Mtb* (DeadMtb; Emerald^+^/TagRFP^−^), and cells containing live *Mtb* (LiveMtb; Emerald^+^/TagRFP^+^). Each sample yielded 100 to 200 million reads, with 0.38%–3.94% mapping to the H37Ra genome in LiveMtb samples, ensuring adequate depth for dual-transcriptomic analysis (Table S1). Three samples (Bystanders_48h_2, DeadMtb_72h_1 and LiveMtb_48h_2) with deviant profiles were excluded (Figures 1B and S1).

The principal-component analysis (PCA) revealed distinct clustering of Mock, Bystander, DeadMtb, and LiveMtb groups, with partial overlap between LiveMtb and DeadMtb, suggesting both shared and unique transcriptional features (Figures 1B and 1C). As Bystander cells may include macrophages that never encountered bacteria, we focused exclusively on LiveMtb and DeadMtb groups for downstream analysis.

Mock cells, representing PMA-differentiated THP-1 macrophages in the absence of infection, were used as the baseline for differential gene expression analysis to distinguish infection-specific changes from background responses. Differentially expressed genes (DEGs) were identified relative to Mock using adjusted *p* value <0.05 and |log_2_FC| > 1, with further classification into LiveMtb- or DeadMtb-associated based on group-specific fold change differences (|ΔLFC| > 0.5) (Figures 1D and 1E). At 48 h, 710 DEGs were found in DeadMtb and 3773 in LiveMtb; at 72 h, these numbers increased to 1060 and 5314, respectively. Only 45 DEGs were shared in DeadMtb across time points, compared to 1826 in LiveMtb (Figure 1F; Tables S2 and S3), highlighting a more robust and sustained transcriptional program in THP-1-derived macrophages harboring live *Mtb*.

### Identification of transcriptomic signatures that bolster macrophage control of *Mtb* infection

**Figure 2:**
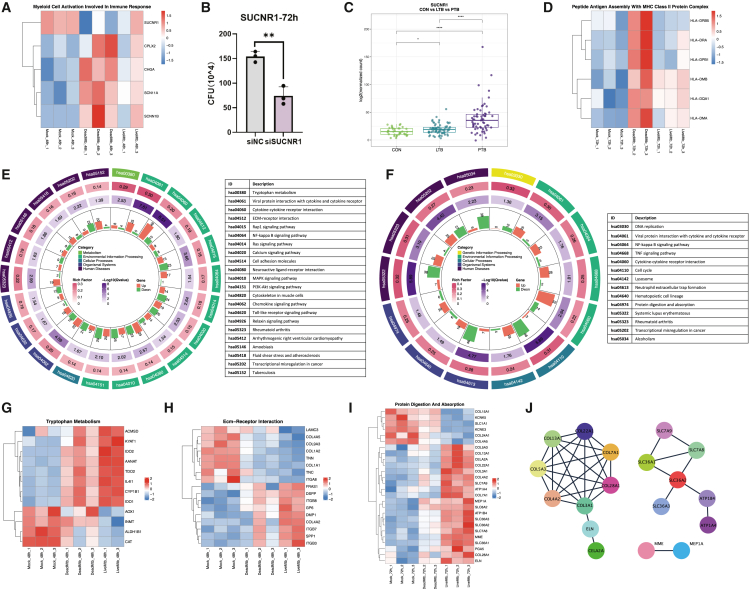
Functional analysis of DEGs in DeadMtb and LiveMtb (A) Heatmap illustrating genes involved in the enriched Gene Ontology (GO) term ‘Myeloid cell activation involved in immune response’ based on DeadMtb DEGs at 48 h p.i. (B) Intracellular H37Rv CFU levels in *SUCNR1*-knockdown THP-1-derived macrophages. Data are represented as mean ± SEM from three independent experiments. (C) Comparison of SUCNR1 expression levels in whole blood from health control (CON), latent TB (LTB) patients, and pulmonary TB (PTB) patients derived from dataset GSE19491. (D) Heatmap showing genes associated with the enriched GO term “Peptide antigen assembly with MHC class II protein complex” based on DeadMtb DEGs at 72 h p.i. (E) Circular plot representing the enriched KEGG pathways identified from LiveMtb DEGs at 48 h and (F) 72 h p.i. From the outer to the inner circles, the plot displays the KEGG pathway ID, the rich factor, the adjusted *p* value, and the number of upregulated and downregulated genes within each pathway. (G) The expression level of genes in GO: tryptophan metabolism. (H) The expression level of genes in GO: ECM-receptor interaction. (I) The expression level of genes in GO: protein digestion and absorption. (J) PPI network of genes in GO: protein digestion and absorption derived from STRING.

The DeadMtb group was used as a proxy in this study to represent effective host control of infection. We next investigated the functional characteristics of DEGs specifically enriched in this group. The results revealed that myeloid cell activation involved in the immune response was enriched at 48 h p.i., containing genes *CHGA*, *CPLX2*, *SCN11A*, *SCNN1B*, and *SUCNR1* (Figure 2A). *CHGA*, a member of the granin family of acidic, soluble glycoproteins stored in secretory granules, served as a precursor of several biologically active peptides with antimicrobial properties.^6^ It also reported to correlate positively with the activation of M1 macrophages and the apoptotic pathway.^7^ Specifically, the downregulated gene *SUCNR1*, which encoded succinate receptor 1, had been reported to promote a local proinflammatory phenotype when deficient in myeloid cells.^8^ As we know, a proinflammatory environment could aid macrophages in killing *Mtb*.^9^ To functionally assess its role in macrophage defense, we performed *SUCNR1* knockdown in THP-1-derived macrophages and observed a significant reduction in intracellular colony-forming unit (CFU) (Figure 2B). In parallel, proinflammatory cytokines (*IL-1β*, *TNF-α*, *IL-6*, *IFN-α*, and *IFN-β*) were measured, only *IL-6* was significantly increased following *SUCNR1* knockdown upon infection (Figure S2). While *IL-6* is a typical pro-inflammatory cytokine, its role during *Mtb* infection is complex, as both protective and detrimental effects have been reported depending on the infection context. Furthermore, analysis of human transcriptomic datasets revealed that *SUCNR1* expression was highest in active TB (ATB) patients, intermediate in individuals with latent TB (LTB) infection, and lowest in healthy controls (CON), suggesting its expression is positively correlated with disease activity (Figure 2C). These findings suggest that *SUCNR1* may act as an immunoregulatory factor that limits macrophage-mediated control of *Mtb*.

We observed robust upregulation of six genes encoding major histocompatibility complex (MHC) class II molecules in the DeadMtb_72h group (Figure 2D), including *HLA-DRA*, *HLA-DRB5*, *HLA-DPB1*, and *HLA-DQA1*, which function as cell surface antigen-presenting receptors. In addition, *HLA-DMA* and *HLA-DMB*, key regulators of antigen processing, were also highly expressed. These intracellular proteins facilitate the removal of CLIP from MHC class II molecules, enabling effective peptide presentation to CD4^+^ T cells.^10^ Although our *in vitro* system lacks adaptive immune components, the coordinated upregulation of these genes suggests that macrophages in the DeadMtb group adopt a transcriptional program characteristic of professional antigen-presenting cells. This MHC class II enrichment likely reflects an enhanced antigen-processing state intrinsic to activated macrophages, rather than a direct effect on T cell priming. Such a primed immune phenotype may contribute to their ability to restrict intracellular *Mtb* replication.

### Identification of transcriptomic signatures in macrophage with persistent *Mtb* infection

The LiveMtb group represents macrophages that harbor viable H37Ra, reflecting both bacterial-driven host modulation and host immune responses. Kyoto Encyclopedia of Genes and Genomes (KEGG) pathway enrichment analysis of LiveMtb DEGs at 48 and 72 h identified common pathways, including cytokine-cytokine receptor interaction, NF-κB signaling, and transcriptional misregulation in cancer (Figures 2E and 2F; Table S4). The top pathway for LiveMtb_48h based on the rich factor was tryptophan metabolism (hsa00380), which was known to be essential for *Mtb* intracellular survival and its ability to cause disease.^11^^,^^12^^,^^13^ Enzymes involved in the two catabolism pathways of tryptophan—kynurenine pathway (*IDO1*, *IDO2*, *TDO2*, *IL4I1*, *ACMSD*, and *KYAT1*), and serotonin pathway (*AANAT* and *CYP1B1*)—were upregulated (Figure 2G). The ECM (extracellular matrix) receptor interaction pathway (hsa04512) was also enriched, which was important for the adhesion of the microbe to the host cells, induction of immune response and aid in the pathogenesis of the disease. Genes such as *SPP1* and integrin proteins (*ITGB3*, *ITGB7*, and *ITGB8*) were upregulated, while ligands for integrins (*TNN* and *TNC*) were downregulated (Figure 2H). Single-cell RNA sequencing (scRNA-seq) of BALF supported our findings, revealing that *SPP1*+ macrophage proportion was significantly higher in TB patients than LTB patients and health control.^14^ SPP1 was a strong chemoattractive and proinflammatory molecule, though its precise mechanism remained unclear. Studies suggested that the arginine-glycine-aspartate (RGD) domain within SPP1 could bind to integrins,^15^^,^^16^ indicating potential interactions among these genes we identified and leading to reduced expression of the ligands. However, the expression of gene in the collagen family (*COL4A5*, *COL9A3*, *COL1A2*, and *COL1A1*) and the laminin family (*LAMC3*) decreased, potentially reflecting matrix remodeling.

Multiple immune and survival-related signaling pathways were also enriched, including the Rap1, Calcium, Ras, MAPK, and PI3K-Akt pathways (Figure 2E). These pathways regulate cytokine production, macrophage activation, cell survival, and granuloma formation. Upregulation of fibroblast growth factor receptor family (*FGF23*, *FGF9*, and *FGF16*) and *VEGFD* in these pathways indicates coordinated immune and repair responses (Figure S3).

Compared to 48 h, the gene sets in the enriched pathways for LiveMtb at 72 h showed more pronounced patterns, with most of the DEGs in each pathway being either upregulated or downregulated (Figure 2F). DNA replication and cell cycle pathways (e.g., hsa03030 and hsa04110) were markedly downregulated, suggesting reduced proliferation. Meanwhile, proinflammatory signaling remained active, with persistent upregulation of NF-κB, Tumor necrosis factor (TNF) pathways, and cytokines (*IL6*, *IL10*, *CCL3*, *CCL4*, *CCL20*, and *CXCL8*) (Figure S4). Interestingly, lysosome-related genes (hsa04142) were significantly enriched only at 72 h, indicating enhanced degradative activity over time. The NET formation pathway (hsa04613) was enriched for downregulated histone genes (*H2A*, *H2B*, *H3*, and *H4*), which may reflect reduced formation of macrophage extracellular traps (METs).^17^ Given that METs can trap and kill bacteria, their suppression might facilitate *Mtb* persistence.^18^

Conversely, the protein digestion and absorption (hsa04974) pathway were upregulated, potentially providing nutrients for bacterial intracellular growth (Figure 2I). This upregulation includes two main categories of genes: amino acid transmembrane transporters (*SLC36A1*, *SLC36A2*, *SLC36A3*, *SLC7A8*, *SLC7A9*, and *SLC1A1*) involved in proline, alanine, and glycine transport; collagen family proteins, which might act as substrates for protein catabolism to provide additional nutrients (Figure 2J).

### Longitudinal profile of gene expression in DeadMtb and LiveMtb macrophages

**Figure 3:**
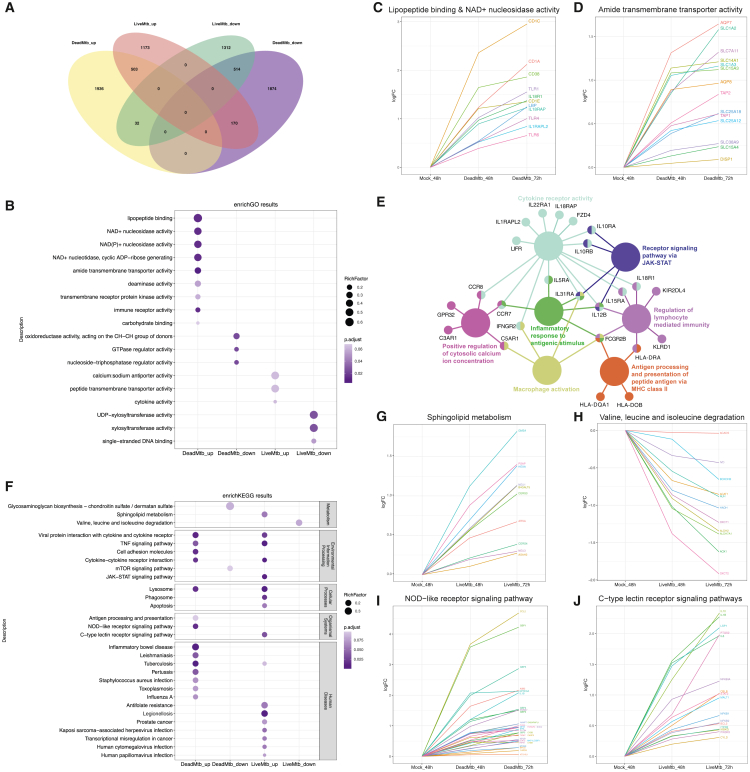
Functional analysis of sustained increase and decrease genes in DeadMtb and LiveMtb (A) Venn diagram of showing genes with sustained increase or sustained decrease across 48 and 72 h in DeadMtb and LiveMtb macrophages. (B) Enriched GO pathways of sustained increase and decrease genes in DeadMtb and LiveMtb. Each dot represents a GO term; the *x* axis shows the sample/cluster, bubble size indicates the number of genes mapped to the term (gene count), and bubble color indicates the adjusted *p* value (p.adjust). Only terms with p.adjust < 0.1 are shown for clarity. (C) The LFC dynamic of genes involved in lipopeptide binding and NAD+ nucleosidase activity. (D) The LFC dynamic of genes involved in amide transmembrane transporter activity. (E) Network representation of GO terms derived from genes annotated to “immune receptor activity.” The network was generated with ClueGO/CluePedia in Cytoscape and shows grouping of related GO terms based on shared genes. Node size is proportional to term significance (-log10 p.adjust), node color indicates functionally grouped clusters, and edges represent shared genes. Representative genes that connect multiple terms (hub genes) are labeled. (F) Enriched GO pathways of sustained increase and decrease genes in DeadMtb and LiveMtb. (G) The LFC dynamic of genes involved in sphingolipid metabolism. (H) The LFC dynamic of genes involved in valine, leucine, and isoleucine degradation. (I) The LFC dynamic of genes involved in C-type lectin receptor signaling pathways. (J) The LFC dynamic of genes involved in NOD-like receptor signaling pathway.

To identify genes potentially associated with infection outcomes, we clustered genes based on expression trends at 48 and 72 h, using mock_48h as a baseline. In DeadMtb_48h, cluster 2 (2471 genes) and cluster 3 (2558 genes) represented sustained increases and decreases, respectively (Figure S5A). Similarly, in LiveMtb_72h, clusters 8 (1846 genes) and 12 (1858 genes) showed these sustained patterns (Figure S5B). DeadMtb and LiveMtb shared 503 upregulated and 514 downregulated genes, with minimal overlap between opposing trends (Figure 3A).

Gene ontology (GO) enrichment analysis revealed no common pathways across the four gene clusters (Figure 3B; Table S4). In DeadMtb, among the genes with sustained increases, the GO with the highest rich factor was lipopeptide binding. It included the CD1 family of transmembrane glycoproteins (*CD1C*, *CD1A*, and *CD1E*) that mediate the presentation of lipid antigens; LBPs (lipopolysaccharide-binding proteins) bind to LPS and interacted with the CD14 receptor, likely playing a role in regulating LPS-dependent monocyte responses; and Toll-like receptors (*TLR1* and *TLR6*) (Figure 3C). NAD+ nucleosidase activity was also enriched, including IL18 receptor (*IL18R1*) and its accessory protein (*IL18RAP*), IL1 receptor accessory protein (*IL1RAPL2*), Toll-like receptors (*TLR1*, *TLR6*, and *TLR4*), and *CD38*, which may be involved in signaling pathways post *Mtb* infection (Figure 3C). The pathway with the lowest *p* value was amide transmembrane transporter activity (Figure 3B). This includes various amino acid transmembrane transport proteins (*SLC1A2*, *SLC1A3*, *SLC15A3*, *SLC15A4*, *SLC25A12*, *SLC25A18*, *SLC14A1*, *SLC7A11*, and *SLC38A9*), as well as channel proteins mediating water and glycerol transport (*AQP7* and *AQP8*) (Figure 3D). Notably, there were also antigen peptide transporters (*TAP1* and *TAP2*), which transport antigens from the cytoplasm to the endoplasmic reticulum for association with MHC class I molecules. In addition, the sustained activation of immune receptor activity encompasses previously identified pathways such as antigen processing and presentation via MHC class II, macrophage activation, positive regulation of cytosolic calcium ion concentration, inflammatory response, lymphocyte-mediated immunity, and receptor signaling through the Janus kinase/signal transducer and activators of transcription (JAK-STAT) pathway (Figure 3E). However, the expression of genes related to GTPase regulator activity and oxidoreductase activity consistently decreased.

For increased genes in LiveMtb, the enriched GO pathways, the activities of calcium:sodium antiporters and peptide transmembrane transporters, did not show significant adjusted *p* values (Figure 3B). However, the genes associated with UDP-xylosyltransferase activity were decreased, including *POGLUT2*, *POGLUT3*, *XXYLT1*, *GXYLT1*, and *RXYLT1*, which were essential for protein O-linked glycosylation. It was known that glycosylation of NOTCH receptors affected NOTCH activation,^19^ and the NOTCH pathway played a role in modulating the immune response during *Mtb* infection.^20^

Regarding the metabolic pathways enriched by mapping genes to KEGG database, genes within sphingolipid metabolism increased continually in LiveMtb (Figures 3F and 3G; Table S4), while that within the degradation of valine, leucine, and isoleucine decreased continually (Figures 3F and 3H; Table S4). Studies have shown that various pathogens, such as bacteria, fungi, and viruses, alter host membrane sphingolipids and their metabolites to manipulate host defense mechanisms, enhancing their survival and pathogenicity.^21^ Additionally, sphingolipid biosynthesis plays a crucial role in phagocytic signaling during *Mtb* entry into the macrophage.^22^ Valine, leucine, and isoleucine were branched-chain amino acids (BCAAs), which could significantly enhance the antibacterial effects of lipopeptides against bacteria.^23^

DeadMtb and LiveMtb shared several consistently activated signaling pathways, including cytokine-cytokine receptor interaction and the TNF signaling pathway (Figure 3F). However, the nucleotide-binding and oligomerization domain (NOD)-like receptor signaling pathway showed a continuous increase in DeadMtb (Figure 3I), while the JAK-STAT and C-type lectin receptor signaling pathways were upregulated in LiveMtb (Figure 3J). Additionally, in DeadMtb, there was an increase in the expression of cell adhesion molecules, notably including MHC class II protein complex assembly genes (HLA family), integrin-binding genes (*ITGAV*, *ITGB7*, *VCAM1*, and *JAM3*), and the Claudins family (*CLDN1*, *CLDN10*, *CLDN12*, and *CLDN14*). Furthermore, genes related to lysosome function consistently increased in both DeadMtb and LiveMtb, whereas genes associated with phagosome and apoptosis pathways showed increased expression exclusively in LiveMtb.

### Distinct transcriptomic profile between intracellular and extracellular *Mtb*

**Figure 4:**
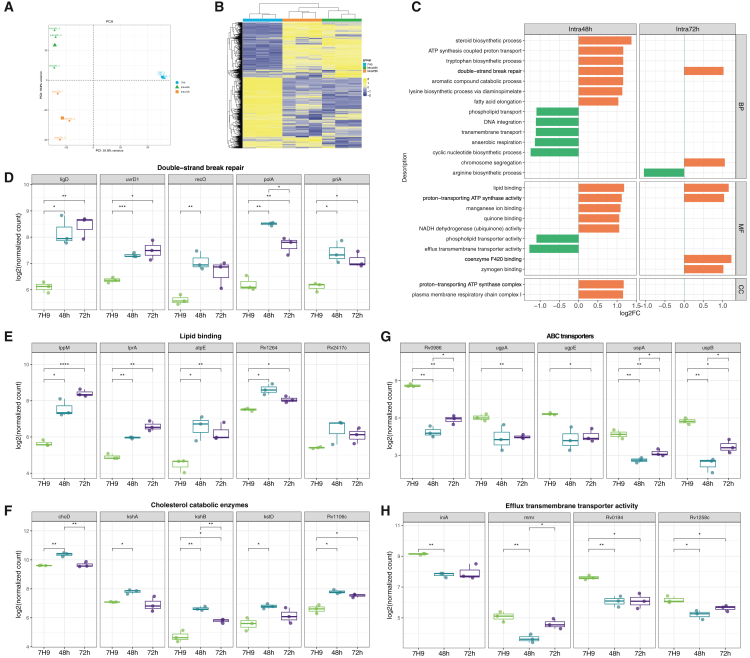
Functional analysis of transcriptome of intracellular *Mtb* (A) PCA plot of gene expression profiles of H37Ra in 7H9, intracellular *Mtb* at 48 and 72 h p.i. (B) Heatmap showing the gene expression profiles among three groups. The colored bars at the top indicate the sample types: blue for H37Ra cultured in 7H9 medium, orange for intracellular H37Ra at 48 h p.i., and green for intracellular H37Ra at 72 h p.i. (C) The LFC of GO terms in intracellular H37Ra compared to those in 7H9. BP, MF, and CC stand for biological process, molecular function, and cellular component, respectively. (D) The comparison of expression level of genes in double-strand break repair among 7H9, intra48h, and intra72h. The *p* value was calculated by *t* test (∗*p* < 0.05, ∗∗*p* < 0.01, ∗∗∗*p* < 0.001, and ∗∗∗∗*p* < 0.0001). (E) The comparison of expression level of genes in lipid binding. (F) The comparison of expression level of genes in cholesterol catabolic enzymes. (G) The comparison of expression level of genes in ABC transporters. (H) The comparison of expression level of genes in efflux transmembrane transporter activity.

Due to the advantages of dual RNA-seq, we obtained transcriptomic data for intracellular live bacteria at both 48 and 72 h (intra48h and intra72h), which had distinct gene expression pattern revealed by PCA analysis (Figure 4A). It highlighted the dynamic adaptation of the bacterium to the intracellular environment. Moreover, genes involved in DNA replication (*dnaA* and *gyrB*), cell division (*ftsZ* and *ftsK*), cell wall synthesis (*murA*), and ribosomal function (*rplA*) were significantly upregulated in intracellular LiveMtb compared to 7H9, consistent with active bacterial replication (Figure S6). Double-strand break repair, lipid binding, and proton-transporting adenosine triphosphate (ATP) synthase activity were upregulated in both intra48h and intra72h *Mtb* (Figure 4B). The antimicrobial activity of macrophages partly stemmed from their production of highly toxic reactive species, including reactive oxygen species and reactive nitrogen species, which could damage *Mtb* DNA to result in single- and double-strand breaks.^24^^,^^25^ Therefore, enzymes involved in DNA repair, such as those for non-homologous end-joining (*ligD* and *uvrD1*) and homologous recombination (*recO*, *polA*, and *priA*), were consistently upregulated. Lipid binding function included five DEGs (*lppM, lprA*, *atpE*, *Rv1264*, and *Rv2417c*). LppM was reported to involve in both phagocytosis and the efficient blockade of phagosome acidification in macrophages, which were crucial processes contributed to the persistence of *Mtb*.^26^ *LprA* was *Mtb* lipoprotein with *TLR2* agonist activity that modulate innate immunity and antigen-presenting cell (APC) functions.^27^ ATP synthase subunit c (*atpE*) was an enzyme that catalyzes the production of ATP from ADP, which could provide energy for *Mtb* persistence. *Rv1264* was an adenylyl cyclase to catalyzes the formation of the second messenger cyclic adenosine monophosphate (cAMP), which regulated the utilization of host lipids by *Mtb* during infection.^28^ *Rv2417c* may function as a fatty acid kinase, binding to long-chain fatty acids like palmitate, and could be involved in lipid transport or fatty acid metabolism.^29^

Furthermore, several metabolism pathways were upregulated in intercellular *Mtb* at 48 h, including steroid biosynthetic process, tryptophan biosynthetic process, aromatic compound catabolic process, and lysine biosynthetic process via diaminopimelate (Figure 4B). Genes involved in the aromatic compound catabolic process included *hsaA*, *hsaB*, *hsaC*, *hsaD*, and *hsaF*. Of these, *hsaA*, *hsaB*, and *hsaD* also played a role in the steroid biosynthetic process, which further involves cholesterol catabolic enzymes like *choD*, *kstD*, *kshA*, *kshB*, and *Rv1106c* (Figure 4F). Like the host (Figure 2D), tryptophan metabolism in *Mtb* was also upregulated, involving four synthases (*trpA*, *trpB*, *trpC*, and *trpG*). Lysine biosynthetic process contained *dapA*, *dapD*, *asd*, *ask*, *lysA*, and *Rv1059*. All these metabolic pathways were essential for intracellular mycobacterial growth.^11^^,^^30^^,^^31^ In contrast, the activity of many transmembrane transporters was downregulated, like phospholipid transporters (*Rv0588*, *yrbE3B*, *yrbE4A*, *yrbE4B*, and *yrbE1A*), ATP-binding cassette (ABC) transporters (e.g., *ugpA*, *ugpE*, *uspA*, *uspB*, *dppC*, *oppD*, *cydD*, and *Rv0986*), and efflux pump (e.g., *tap*, *iniA*, *mmr*, and *Rv0194*). *Rv0986* has been reported to play a role in the ability of *Mtb* to bind to host cells.^32^

At 72 h, *Mtb* exhibited a characteristic upregulation of chromosome segregation (*ftsK*, *parB*, *scpA*, *xerC*, and *Rv1701*), while the arginine biosynthetic process decreased (*argC*, *argG*, and *Rv2141c*). These results suggest that the regulation of arginine metabolism by *Mtb* is highly context-dependent, potentially reflecting distinct nutrient availability or host metabolic pressures during infection.

### Correlation analysis between *Mtb* and host GO functional pathways

**Figure 5:**
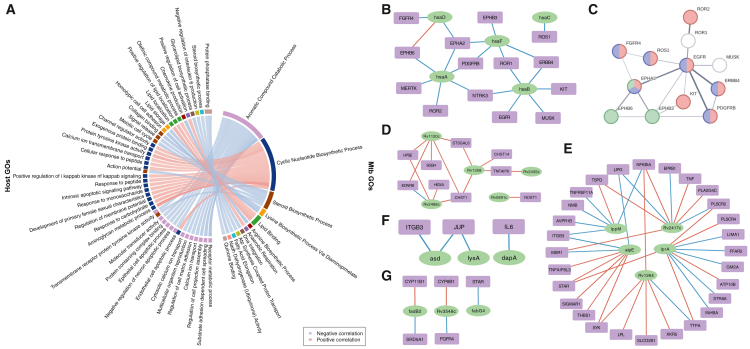
Correlation analysis of host GOs and *Mtb* GOs (A) The significant correlation between host GOs and *Mtb* GOs. (B) The *Mtb* genes related to aromatic compound catabolic process and the host genes related to transmembrane receptor protein tyrosine kinase activity. (C) PPI network of host genes listed in (B). Red, purple, and green indicate genes associated with GO terms: positive regulation of MAP kinase activity, regulation of ERK1 and ERK2 cascade, and ephrin receptor activity, respectively. (D) The *Mtb* genes related to cyclic nucleotide biosynthetic process and the host genes related to aminoglycan metabolic process. (E) The *Mtb* genes related to lipid binding and the host genes related to lipid localization. (F) The *Mtb* genes related to lysine biosynthetic process via diaminopimelate, and the host genes related to cell adhesion. (G) The *Mtb* genes related to fatty acid elongation and the host genes related to steroid biosynthetic process.

A major advantage of dual RNA-seq is the simultaneous capture of host and pathogen transcriptomes within the same infection context. To explore potential cross-species functional interactions, we performed correlation analysis between host and *Mtb* GO pathways using Gene set variation analysis (GSVA)-transformed expression matrices at 48 h and 72 h post-infection. Pathway pairs with strong correlations (|*r*| ≥ 0.8, *p* < 0.001) were retained (Figure 5A).

Aromatic compound catabolism in *Mtb* was among the most notable findings, showing negative correlations with several host immune-related pathways, including membrane receptor activity, calcium signaling, GPCR signaling, and cell matrix adhesion. Notably, it also inversely correlated with apoptotic signaling. These aromatic compounds (such as phenylalanine, tyrosine, and tryptophan) are not only bacterial carbon sources but also linked to host steroid biosynthesis. Accordingly, the metabolic activity of key *Mtb* catabolic enzymes (*hsaA/B/C/D/F*) showed strong inverse correlation with several host receptor tyrosine kinases, including *EGFR*, *ERBB4*, *PDGFRB*, *KIT*, *FGFR4*, and *FLT3* (Figure 5B), all of which are core components of the Rap1, Ras, MAPK, and PI3K-Akt signaling pathways involved in cell proliferation, survival, and immune modulation(Figure 5C). Additionally, Ephrin receptor family members (*EPHA2*, *EPHB6*, and *EPHB3*) exhibited strong inverse correlation with the same aromatic metabolic genes. Consistent with the RNA-seq results, flow cytometry analysis confirmed that EPHB6 expression was lower in macrophages with live *Mtb* compared to the ones with dead *Mtb* (Figure S7). Prior studies have implicated Ephrin signaling in cholesterol uptake regulation,^33^ and hsaA has also been linked to cholesterol catabolism in bacteria. This suggests that *Mtb* aromatic metabolism may disrupt host signaling while leveraging host lipid pathways to support its survival.

In contrast, *Mtb* cyclic nucleotide biosynthesis, especially cAMP-related activity, positively correlated with host immune pathways such as peptide and monosaccharide responses and aminoglycan metabolism, the latter showing the strongest statistical association. *Mtb* adenylyl cyclases (*Rv1120c* and *Rv2488c*) were associated with host genes *SGSH*, *HPSE*, and *HEXA*, involved in lysosomal degradation of glycosaminoglycans (Figure 5D), implying that *Mtb* may modulate host lysosomal function via cAMP signaling.

Genes involved in lipid-binding in *Mtb* (*lppM*, *lprA*, *atpE*, *Rv1264*, and *Rv2417c*) showed consistent positive correlations with host genes related to lipid transport and metabolism (*LPL*, *TTPA*, *TSPO*, *LIPG*, and *PLSCR2*) (Figure 5E), suggesting co-adaptation for lipid resource utilization.

Antagonistic correlations were also noted. *Mtb* lysine biosynthesis (*asd* and *lysA*) inversely correlated with host adhesion genes (*ITGB3* and *JUP*) (Figure 5F), while fatty acid elongation genes (*fadB2*, *fabG4*, and *Rv3548c*) showed negative correlations with host steroid biosynthetic genes (*CYP11B1*, *CYP8B1*, *SRD5A1*, *FGFR4*, and *STAR*) (Figure 5G), indicating potential *Mtb* strategies to impair host endocrine signaling and tissue integrity.

Several additional correlations revealed potential antagonistic interactions. The lysine biosynthesis via diaminopimelate pathway in *Mtb* negatively correlated with host cell adhesion, with *Mtb* genes *asd* and *lysA* showing inverse correlation with *ITGB3* (integrin family) and *JUP* (junction plakoglobin) (Figure 5F), both important for maintaining macrophage adhesion and barrier integrity. Similarly, the fatty acid elongation pathway in *Mtb* was negatively correlated with the host steroid biosynthetic process. Specifically, fadB2 correlated inversely with *CYP11B1* and *SRD5A1*, *Rv3548c* with *CYP8B1* and *FGFR4*, and *fabG4* with *STAR* (Figure 5G), suggesting that increased *Mtb* fatty acid metabolism may suppress host steroid hormone synthesis, potentially disrupting immune-endocrine signaling.

## Discussion

This study combines dual RNA-sequencing with fluorescently labeled *Mtb* to investigate macrophage responses and host-pathogen interactions from two perspectives. First, FACS-based separation of THP-1-derived macrophages harboring live or dead H37Ra enabled single-cell resolution of immune states. DeadMtb macrophages reflect effective bacterial clearance, while LiveMtb cells represent immune evasion and persistent infection. Differential gene expression between these groups identified host transcriptional programs associated with bactericidal activity versus bacterial persistence. Second, time-resolved transcriptomic profiling of both host and pathogen allowed correlation analysis across functional pathways, offering insight into potential cross-regulatory mechanisms. Caution is warranted in interpreting these relationships, and further mechanistic studies are needed to validate the causal roles of key genes and pathways.

Macrophages in the DeadMtb group exhibited strong immune activation, characterized by the upregulation of macrophage activation markers, inflammatory cytokine responses, and antigen presentation pathways, sustained across 48 and 72 h (Figures 2A, 2B, 3B, and 3F). In addition to classical MHC class II antigen presentation, these macrophages showed enhanced presentation of lipid antigens through CD1 molecules (*CD1A*, *CD1C*, and *CD1E*) (Figure 3C), which recognize mycobacterial glycolipids and broaden immune detection.^34^ Elevated lysosomal gene expression (Figure 3E) further supported a heightened degradative capacity, enabling antigen generation from both peptides and lipids, consistent with effective intracellular clearance mechanisms.^2^^,^^35^ Although one replicate of the DeadMtb_48h group (DeadMtb_48h_2) displayed a transcriptional pattern closer to LiveMtb (Figures 2G and 2H), this likely reflects biological heterogeneity or minor technical variation. Overall, the consistent clustering of the other replicates supports the observed immune activation patterns. It should be also noted that the DeadMtb population may include macrophages that phagocytosed non-viable bacilli from the inoculum, in addition to those that actively killed bacteria.

In contrast, LiveMtb macrophages displayed persistent activation of cytokine-cytokine receptor and NF-κB signaling (Figures 2E, 2F, and 3F), indicative of chronic inflammation. Intracellular *Mtb* responded by upregulating DNA repair genes (e.g., *uvrD1* and *recO*) and remodeling metabolism to counter host stress (Figure 4D). Alterations in ECM and adhesion pathways were evident, with upregulation of SPP1 and integrins (e.g., ITGB3) potentially enhancing macrophage recruitment. However, downregulation of integrin ligands (e.g., TNC) suggests that *Mtb* may disrupt ECM signaling to facilitate persistence (Figure 2H).

Amino acid metabolism was notably altered in LiveMtb group. Tryptophan catabolism via the kynurenine pathway was enhanced (Figure 2G), consistent with its known role in immunosuppression and inhibition of antigen presentation.^36^ Upregulation of protein digestion and absorption pathways may reflect host attempts to compensate metabolically or bacterial exploitation of nutrient resources (Figure 2I).

Transcriptomic profiling of intracellular *Mtb* revealed upregulation of genes involved in DNA repair, lipid binding (*lppM*, *lprA*, and *Rv2417c*), and ATP production (*atpE*) (Figures 4B and 4E), all contributing to persistence within macrophages.^37^ Downregulation of ABC and efflux transporters (Figures 4G and 4H) may reflect metabolic adaptation to minimize antigen exposure and energy consumption.

Among the most notable findings from the correlation analysis, *Mtb* aromatic compound catabolism (*hsaA-F*), responsible for degrading phenylalanine, tyrosine, and tryptophan, was negatively correlated with host receptor tyrosine kinase signaling (*EGFR*, *PDGFRB*, *KIT*, and *FGFR4*) and Ephrin receptors (*EPHA2*, *EPHB3*, and *EPHB6*) (Figure 5B). These receptors are critical for immune activation, lipid signaling, and steroid biosynthesis.^38^ It suggests that *Mtb* may leverage aromatic metabolism to suppress host immune recognition while exploiting lipid and cholesterol intermediates for intracellular persistence, a hypothesis supported by known roles of both Ephrin signaling and *hsaA-F* in cholesterol regulation.

Beyond aromatic metabolism, our analysis revealed additional layers of metabolic cross-talk, including the positive correlation between *Mtb* cyclic nucleotide biosynthesis and host lysosomal and glycosaminoglycan degradation pathways, potentially reflecting bacterial modulation of host signaling through cAMP.^39^ Coordinated expression between *Mtb* lipid metabolism genes and host lipid transporters, as well as inverse associations between *Mtb* lysine or fatty acid biosynthesis and host cell adhesion and steroid pathways, further suggest that *Mtb* actively reprograms the host intracellular environment to balance immune evasion, nutrient acquisition, and structural modulation. Together, these findings provide a comprehensive view of host-pathogen transcriptional dynamics during *Mtb* infection and highlight metabolic pathways as key interfaces in immune modulation and bacterial survival.

### Limitations of the study

It should be noted the dual RNA-seq experiments in this study were performed using the attenuated strain H37Ra, which may miss some virulence-associated genes compared with H37Rv. Nonetheless, key host transcriptional signatures and metabolic interactions were validated with H37Rv (e.g., *SUCNR1*), supporting the robustness of our conclusions, though future studies with H37Rv will be needed to fully capture virulence specific host-pathogen interactions.

## Resource availability

### Lead contact

Further information and requests for resources and reagents should be directed to and will be fulfilled by the lead contact, Xinchun Chen (chenxinchun@szu.edu.cn).

### Materials availability

This study did not generate new unique reagents.

### Data and code availability

• The raw sequencing datasets for the study are available in the NCBI Sequence Read Archive (SRA) repository, under the Bioproject with accession code PRJNA1150773 (http://www.ncbi.nlm.nih.gov/bioproject/1150773).
• Pepline used in this study have been deposited in https://github.com/shichenyan/Dual-RNA-seq-using-fluorescent-Mtb.
• Any additional information required to reanalyze the data reported in this paper is available from the lead contact upon request.

## Acknowledgments

This work was supported by the Young Scientists Fund of the 10.13039/501100001809National Natural Science Foundation of China (grant no. 82202574) and Shenzhen Medical Research Fund (grant no. A2304001).

## Author contributions

C.S. designed the project and performed the bioinformatics analysis with support from Z.L.; X.L., T.W., D.C., Y.W., and N.C. conducted the wet lab experiments; C.S. drafted the manuscript; and L.H., Y.C., and X.C. reviewed the manuscript.

## Declaration of interests

The authors declare no competing interests.

## STAR★Methods

### Key resources table

**Table:** 

REAGENT or RESOURCE	SOURCE	IDENTIFIER
**Antibodies**		

APC anti-human EPHB6 Antibody	BioLegend	Cat#384305; RRID: AB_3068073

**Bacterial and virus strains**		

*M. tuberculosis* H37Ra with dual-color reporter	Lab stock	provided by Christopher M. Sassetti, University of Massachusetts Medical School
*M. tuberculosis* H37Ra	Lab stock	N/A
*M. tuberculosis* H37Rv	Lab stock	N/A

**Chemicals, peptides, and recombinant proteins**		

Tetracycline	MedChemExpress	Cat#64-75-5
Paraformaldehyde	Sigma-Aldrich	Cat#30525-89-4
TRIzol Reagent	Thermo Fisher	Cat#15596026
Lipofectamine RNAiMA	Invitrogen	Cat#13778075
Phorbol 12-myristate 13-acetate (PMA)	Thermo Fisher	Cat#16561-29-8
Human IL-6 ELISA Kit	LiankeBio	Cat#EK106/2-96
Human TNF-α ELISA kit	HUABIO	Cat#EH0002
Human IL-1β ELISA kit	LiankeBio	Cat#EK101B-96
Human IFN-β ELISA kit	LiankeBio	Cat#EK1236-96
Human IFN-α ELISA kit	LiankeBio	Cat#EK199HS-96
Total RNA Kit	Omega	Cat#R6834
SYBR Green PCR Master Mix	BIO-RAD	Cat#1725271
Anhydrotetracycline (ATC)	Thermo Fisher	Cat#13803-65-1
RPMI-1640	Gibco	Cat#11875093
Ampure XP Beads	Beckman Coulter	Cat#A63880

**Deposited data**		

Raw data	This study	PRJNA1150773
Blood Transcriptional Profiles in Human Active and Latent Tuberculosis	Berry et al.^40^	GEO: GSE19491

**Experimental models: cell lines**		

Human: THP-1 cells	Cell Bank of the Chinese Academy of Sciences (Shanghai, China)	SCSP-567

**Oligonucleotides**		

*SUCNR1*-siRNA-1*:* GGAGAUCACUUCAGGGACATT	This paper	N/A
*SUCNR1*-siRNA-2: UGUCCCUGAAGUGAUCUCCTT	This paper	N/A
*SUCNR1* forward primer: GGAGACGTGCTCTGCATAAG	This paper	N/A
*SUCNR1* reverse primer: AGGTGTTCTCGGAAAGGATACTT	This paper	N/A

**Software and algorithms**		

Trim Galore	Krueger et al.^41^	https://github.com/FelixKrueger/TrimGalore
Bowtie2	Langmead et al.^42^	https://doi.org/10.1038/nmeth.1923
HISAT2	Kim et al.^43^	https://doi.org/10.1038/nmeth.3317
HTseq	Putri et al.^44^	https://doi.org/10.1093/bioinformatics/btac166
Limma	Ritchie et al.^45^	https://doi.org/10.1093/nar/gkv007
clusterProfiler	Yu et al.^46^	https://www.bioconductor.org/packages/release/bioc/html/clusterProfiler.html
Cytoscape	Shannon et al.^47^	https://cytoscape.org/
Mfuzz	Kumar et al.^48^	https://doi.org/10.6026/97320630002005
GSVA	Hanzelmann et al.^49^	https://doi.org/10.1186/1471-2105-14-7
psych	Revelle et al.^50^	https://cran.r-project.org/web/packages/psych/
ggplot2	Wickham et al.^51^	https://ggplot2.tidyverse.org/
circlize	Gu et al.^52^	https://doi.org/10.1093/bioinformatics/btu393
ComplexHeatmap	Gu et al.^53^	https://doi.org/10.1093/bioinformatics/btw313
GraphPad Prism v8	N/A	https://www.graphpad.com/scientific-software/prism/

**Other**		

Raw data of Dual RNA-seq	This paper	http://www.ncbi.nlm.nih.gov/bioproject/1150773
Pepline of Dual RNA-seq analysis	This paper	https://github.com/shichenyan/Dual-RNA-seq-using-fluorescent-Mtb

### Experimental model and study participant details

#### Bacteria strains

*Mycobacterium tuberculosis* H37Rv, wild-type H37Ra, and a dual-color reporter strain of H37Ra that carries a constitutively expressed Emerald fluorescent protein (green) and a tetracycline-inducible TagRFP fluorescent protein (red) were used in this study. The reporter strain was kindly provided by Christopher M. Sassetti at the University of Massachusetts Medical School. All strains were cultured either in Middlebrook 7H9 broth supplemented with 0.2% (vol/vol) glycerol, 0.25% (vol/vol) Tween-80, and 10% OADC or on Middlebrook 7H10 plates supplemented with 0.5% glycerol and 10% OADC.

#### Cell lines

The human monocytic THP-1 cell line was maintained at 37 °C in a humidified atmosphere containing 5% CO_2_ in RPMI 1640 medium supplemented with 10% fetal bovine serum (FBS), 2% L-glutamine, and 1% penicillin-streptomycin. For differentiation into macrophages prior to infection, THP-1 cells were treated with 40 ng/mL phorbol 12-myristate 13-acetate (PMA) for 48 hours. The human monocytic THP-1 cell line was purchased from the Cell Bank of the Chinese Academy of Sciences (Shanghai, China; Cat. No. SCSP-567). According to the supplier documentation, the cell line was authenticated by short tandem repeat (STR) profiling and tested negative for mycoplasma contamination prior to shipment.

### Method details

#### Mtb infection and cell sorting

The PMA-differentiated THP-1 macrophages were infected with an *Mtb* strain H37Ra (MOI = 10) harboring a dual-color reporter. At 24h and 48h post infection, tetracycline (500 ng/ml) (catalog no. 64-75-5; MedChemExpress) was added to the medium, and macrophages were harvested after 24 h of incubation, fixed with 4% paraformaldehyde (PFA), and analyzed using a BD FACS Canto II (BD Biosciences), as previously described.^54^ Mock controls were included at both 48h and 72h, consisting of PMA-differentiated THP-1 macrophages treated under identical conditions (including tetracycline addition and fixation) but without *Mtb* infection. Each condition was performed with three independent biological replicates. The dual-fluorescent reporter strain used to distinguish live and dead *Mtb* has been previously validated,^55^ where CFU assays confirmed that no culturable bacteria remained in the DeadMtb population.

#### RNA extraction for dual RNAseq

Trizol was added to the sorted cells and incubated at room temperature for 5 minutes to fully dissociate the host cell nucleoprotein complexes. The samples were then centrifuged for 20 minutes to pellet the intact *Mtb* cells. Subsequently, 80% of the Trizol solution (containing host RNA) was carefully removed and transferred into RNase-free tubes for later use. Fresh Trizol and 0.1 mm zirconium oxide/silica beads were then added to the tubes containing the *Mtb* pellet, followed by mechanical lysis using a BeadBeater. Depending on the quantity of sorted cells, a portion of the host RNA (typically >50% of the volume) was then added back to the tube containing the bacterial RNA. The mixture was then processed according to the Trizol protocol for RNA extraction.

#### rRNA removal library construction and sequencing

The concentration of RNA samples was assessed using the Agilent 2100 Bioanalyzer (Agilent RNA 6000 Nano Kit), where the RNA Integrity Number (RIN), 28S/18S ratio, and fragment size were measured. The purity of the samples was determined using a NanoDrop™ spectrophotometer. The digestive system was prepared and incubated at the appropriate temperature for a specific period in a Thermomixer. DNase I was then used to digest any DNA fragments present in the total RNA samples. Magnetic beads were employed to purify and recover the reaction products, which were subsequently dissolved in DEPC-treated water.

Next, a portion of the total RNA samples was taken, and rRNA was removed using either the RNase H or Ribo-Zero method. The resulting samples were treated with Fragmentation Buffer and thermally fragmented in a PCR machine to generate RNA fragments of 130-160 nucleotides. First-strand cDNA synthesis was performed using a First Strand Mix, followed by the addition of a Second Strand Mix to produce second-strand cDNA. The reaction products were purified using magnetic beads. The purified fragmented cDNA was then subjected to end repair using End Repair Mix, followed by the addition of A-Tailing Mix, which was thoroughly mixed by pipetting and incubated. The adenylated 3′ ends of the DNA were ligated to RNA Index Adapters using Ligation Mix, followed by additional pipetting and incubation. Several rounds of PCR amplification were conducted using PCR Mix to enrich the cDNA fragments. The PCR products were purified with Ampure XP Beads (AGENCOURT). The double-stranded PCR products were then heat-denatured and circularized using a splint oligo sequence to form single-stranded circular DNA (ssCir DNA), which served as the final library. The library was amplified using phi29 polymerase to create DNA nanoballs (DNBs), each containing over 300 copies of a single molecule. The DNBs were loaded onto a patterned nanoarray, and single-end 50 base pair (or paired-end 100 base pair) reads were generated using combinatorial Probe-Anchor Synthesis (cPAS). Finally, the library was sequenced on the DNBSEQ platform.

#### RNA-seq analysis

Raw sequencing reads were analyzed following the protocol described by Pisu et al. The workflow is as follows: First, the raw reads were quality filtered using Trim Galore (v. 0.6.4)^41^ to remove adapter sequences and low-quality reads. The filtered reads were then aligned to rRNA sequences using Bowtie2 (v. 2.3.5.1, --very-sensitive mode),^42^ and rRNA reads were removed. The remaining reads were further aligned to the H37Ra genome (GCF_000016145.1) using Bowtie2, and the reads were separated into two categories: *Homo sapiens* reads and H37Ra reads. Each set of reads was then aligned to GRCm38.94 and GCF_000016145.1 genomes using HISAT2 (v. 2.2.1),^43^ respectively. The number of reads mapped to each gene was quantified using htseq-count (v. 0.6.1),^44^ resulting in the transcript expression levels. The pipeline was provided in https://github.com/shichenyan/Dual-RNA-seq-using-fluorescent-Mtb/blob/main/DualRNAseq_pipeline.sh.

#### siRNAs transfection and CFU

PMA-differentiated THP-1 macrophages were transfected with *SUCNR1-specific siRNAs* (GGAGAUCACUUCAGGGACATT and UGUCCCUGAAGUGAUCUCCTT) using Lipofectamine RNAiMAX (Invitrogen), according to the manufacturer’s protocol and as previously described.^56^ Scrambled siRNA was used as a negative control. Knockdown efficiency was assessed by qPCR 36 to 48 hours post-transfection (Figure S2A). At 48 hours post transfection, the macrophages were infected with H37Rv (MOI = 10) for 6 hours, followed by three washes with PBS to remove extracellular bacteria. At 72 hours post infection, the cells were lysed with 0.1% SDS, and serial dilutions were plated on 7H10 plates. The numbers of colony-forming units (CFU) were counted after incubation at 37°C for 2 - 4 weeks, as previously described.^56^

#### SUCNR1 analysis in human whole blood transcriptomic datasets

Publicly available gene expression data from whole blood of healthy controls (HC), latent TB (LTB), and active TB (ATB) patients (GSE19491^40^) were downloaded and processed. Sample metadata were curated to assign groups, remove samples with other diseases, and extract relevant patient and timepoint information. *SUCNR1* expression was extracted, and comparisons across HC, LTB, and ATB were performed. Expression levels were visualized using boxplots, and statistical significance was assessed using the Wilcoxon rank-sum test with multiple group comparisons.

#### RNA extraction and quantitative PCR

Total RNA was extracted from THP-1 macrophages using a Total RNA Kit (Omega), and quantitative PCR was performed using a 7500 Fast Real-Time PCR System (Thermo Fisher Scientific) with SYBR Green PCR Master Mix (BIO-RAD), according to the manufacturer’s instructions and as previously described.^57^ The primers obtained from the PrimerBank were as follows: *SUCNR1* (forward: 5′-GGAGACGTGCTCTGCATAAG-3′; reverse: 5′-AGGTGTTCTCGGAAAGGATACTT-3′). Cycling conditions were as follows: 95 °C for 10 min, followed by 40 cycles of 95 °C for 15 s and 60 °C for 34 s.

#### Measurement of inflammatory cytokines

The concentrations of IL1β, TNF-α, IL-6, IFN-α and IFN-β in culture supernatants of THP-1-derived macrophages 72 h post-infection with H37Rv (MOI = 5) following SUCNR1 knockdown or scrambled control were determined using an enzyme-linked immunosorbent assay (ELISA) kit (Liankebio, China) according to the manufacturer’s instructions.

#### Flow cytometry analysis of EPHB6

PMA-differentiated THP-1 macrophages were infected with H37Ra (MOI=10). Six hours post-infection, the culture medium (RPMI-1640) was replaced. After 48 h of infection, anhydrotetracycline (ATC) was added at a final concentration of 500 ng/mL to induce expression Following 24 h of induction, cells were harvested, washed twice with PBS, resuspended in FACS buffer, and stained with anti-EPHB6 antibody (BioLegend, Cat#384305). EPHB6 expression was analyzed using flow cytometry.

### Quantification and statistical analysis

The differential gene expression analysis between groups was carried out in R using the limma pipeline,^45^ which fitting a linear model for each gene and then performing empirical Bayes statistics. The DEG were identified using an absolute Log2FC greater than 1 and an adjusted p-value of less than 0.05 as the selection criteria. The GO and KEGG enrichment analysis of host DEGs were conducted using clusterProfiler^46^ package in R. The protein-protein interaction network was created in Cytoscape^47^ using the STRING app (1.4.1). Temporal trend analysis of gene expression changes was performed using the Mfuzz package.^48^

The gene expression matrix for *Mtb* was transformed into a GO expression matrix using the GSVA package,^49^ based on the *Mtb* GO database derived from the DAVID database.^58^ For host genes, the transformation was based on the C5 category of the MSigDB database. Differentially expressed GO functions were identified using the limma pipeline, applying the same thresholds as for DEGs.

Pearson correlations between the *Mtb* GO and LiveMtb GO matrices were calculated using the psych package,^50^ with p-values adjusted using the Holm method. The filtering criteria were set to a correlation coefficient of ≥ 0.8 and an adjusted p-value of < 0.001, and delete GOs belonging to cellular component. Gene correlations within the filtered correlated GOs were calculated using the same method and visualized in Cytoscape. Additional visualizations in the study were created using the ggplot2,^51^ circlize,^52^ and ComplexHeatmap^53^ packages.

Statistical analyses were performed using GraphPad Prism version 8 (GraphPad Software Inc.), with statistical significance of differences between groups was determined using a student’s unpaired t test.

## Supplemental information

Document S1. Figures S1–S7

Table S1. Number of reads in all samples

Table S2. The DEGs at 48 h

Table S3. The DEGs at 72 h

Table S4. The KEGG enrichment results of LiveMtb DEGs

Table S5. The GO enrichment results of consistently increased and decreased genes

Table S6. The KEGG enrichment results of consistently increased and decreased genes

Table S7. Significantly differential GO function between intracelluar and extracellular growth *Mtb*
