# Supplementary material for: Dual transcriptomic analysis unraveling the immune landscape and host-pathogen interactions during Mycobacterium tuberculosis infection
Source: iScience. 2025 Nov 17;28(12):114102. doi: 10.1016/j.isci.2025.114102 (PMC12765389; doi:10.1016/j.isci.2025.114102)
Supplement: Document S1. Figures S1–S7 [file mmc1.pdf]

iScience, Volume *28*

Supplemental information

Dual transcriptomic analysis unraveling the immune landscape and host-pathogen interactions during *Mycobacterium tuberculosis* infection

Chenyan Shi, Xiaoqian Liu, Dan Chen, Tong Wang, Yu Wang, Ningjian Cai, Zhaodong Li, Yunlong Hu, Yi Cai, and Xinchun Chen

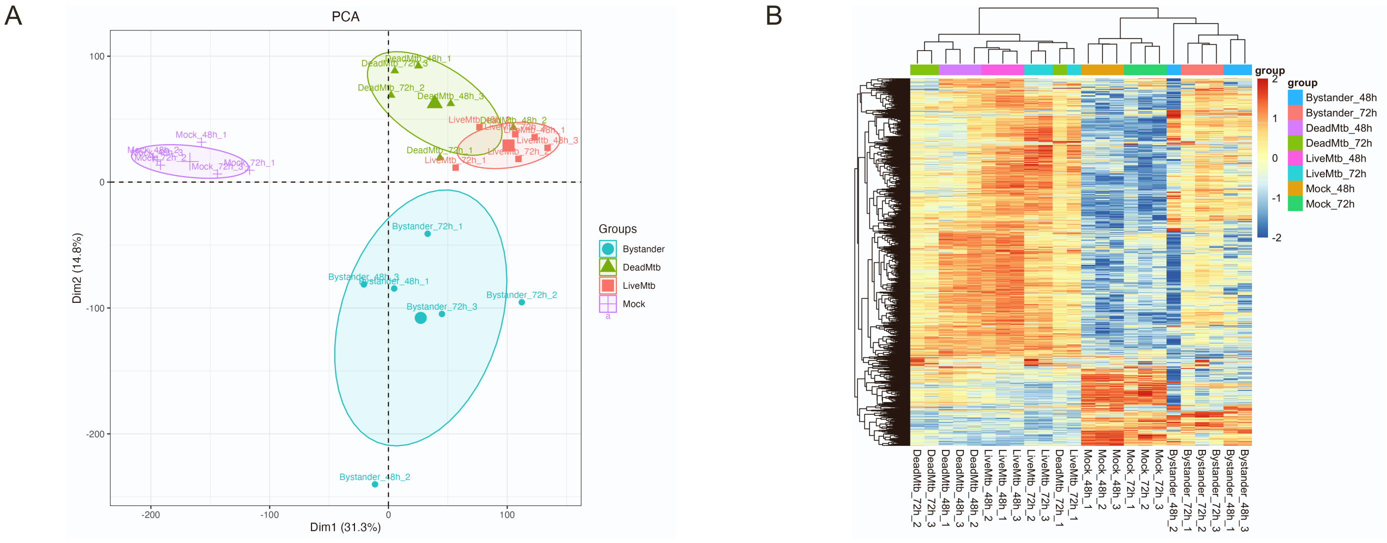

Figure S1. The overview of the transcriptome in all samples. A) The PCA plot of all samples. B) The heatmap of top 1000 genes based on the standard deviation of expression among samples.

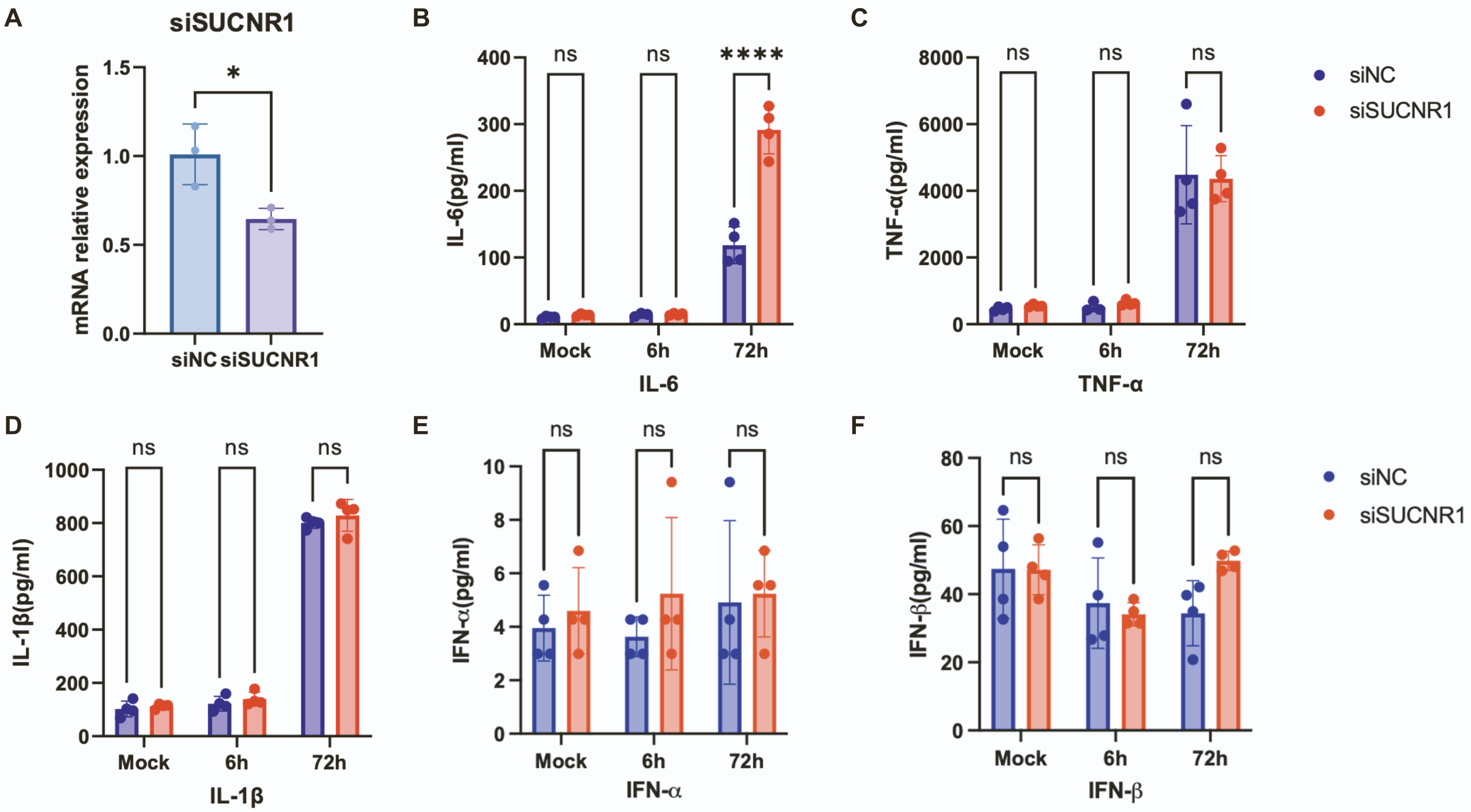

Figure S2. SUCNR1 knockdown in THP-1-derived macrophages and its impact on pro-inflammatory cytokine production upon H37Rv infection. (A) qPCR analysis showing the knockdown efficiency of SUCNR1 in THP-1-derived macrophages transfected with SUCNR1-targeting siRNA (siSUCNR1) compared with scrambled control (siNC). (B–F) ELISA measurements of cytokine levels (IL-6, TNF-α, IL-1β, IFN-α and IFN-β) in the culture supernatants of THP-1-derived macrophages 72 h post-infection with H37Rv (MOI = 5) following SUCNR1 knockdown or scrambled control. Data are presented as mean ± SD from four independent experiments. Statistical significance was determined using t-test(*p < 0.05, **p < 0.01, ***p < 0.001, ****p < 0.0001, ns: not significant).

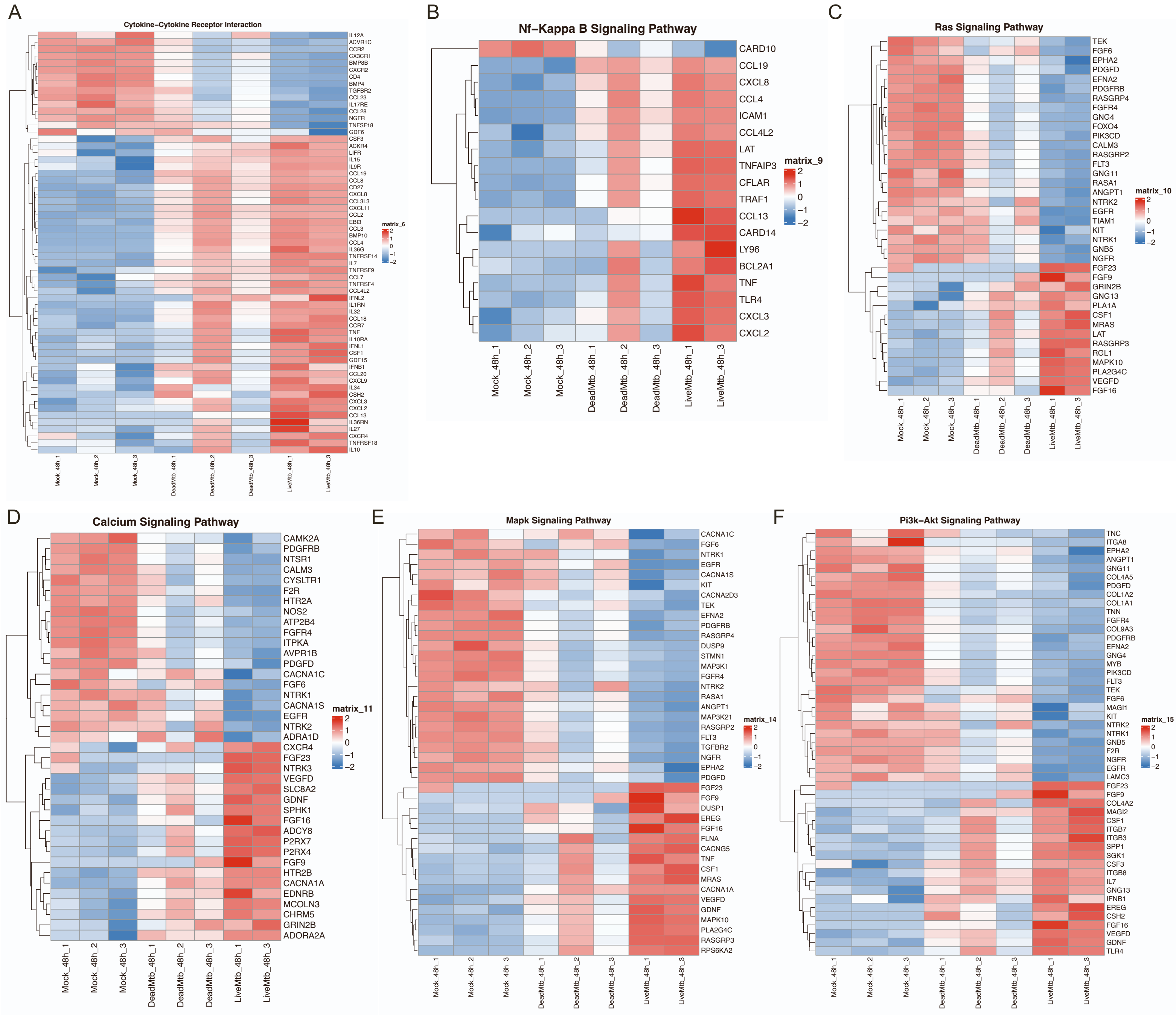

Figure S3. The expression heatmap of genes involved in the enriched KEGG pathways from LiveMtb_48h DEGs. A) Genes involved in Cytokine–Cytokine Receptor Interaction. B) Genes involved in NF-κB signaling pathway. C) Genes involved in Ras signaling pathway. D) Genes involved in Calcium signaling pathway. E) Genes involved in MAPK signaling pathway. F) Genes involved in Pi3k-Akt signaling pathway.

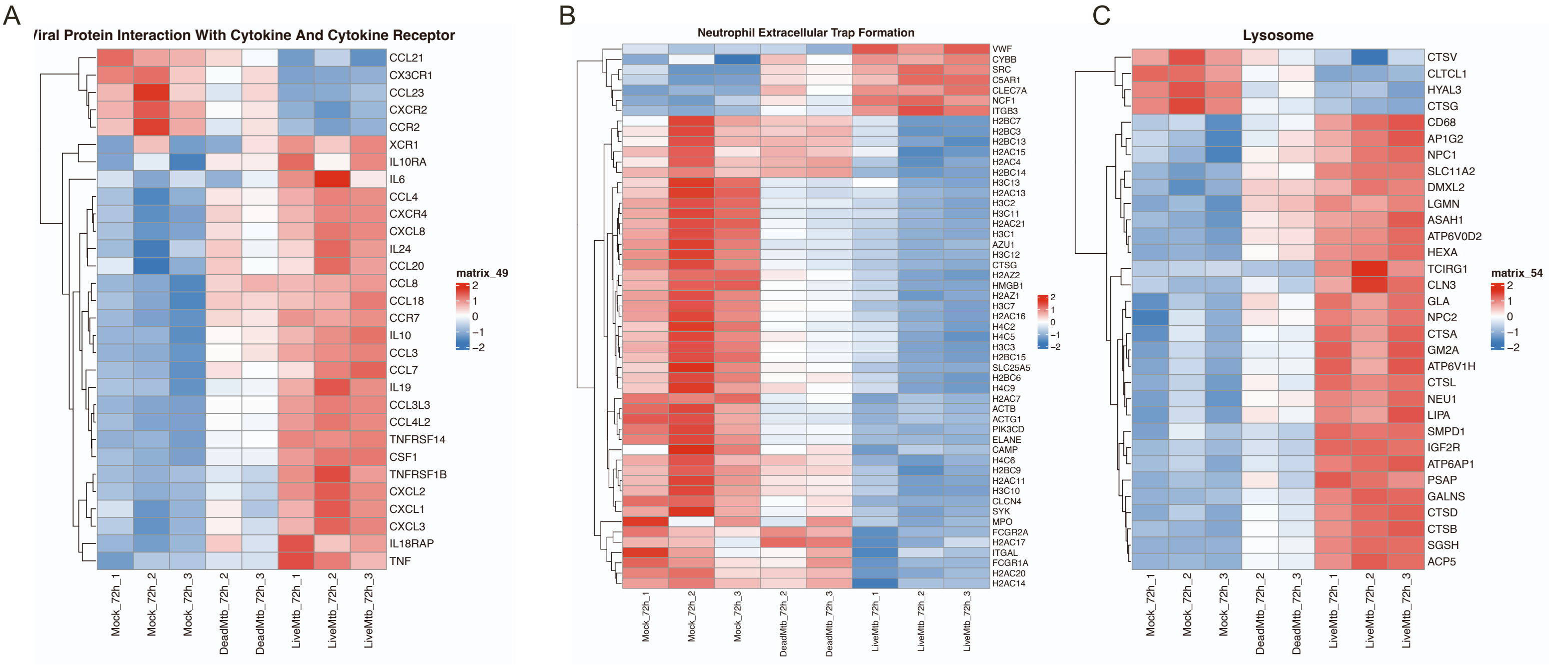

Figure S4. The expression heatmap of genes involved in the enriched KEGG pathways from LiveMtb_72h DEGs. A) Genes involved in Viral Protein Interaction With Cytokine And Cytokine Receptor. B) Genes involved in Neutrophil Extracellular Trap Formation. C) Genes involved in Lysosome.

A

B

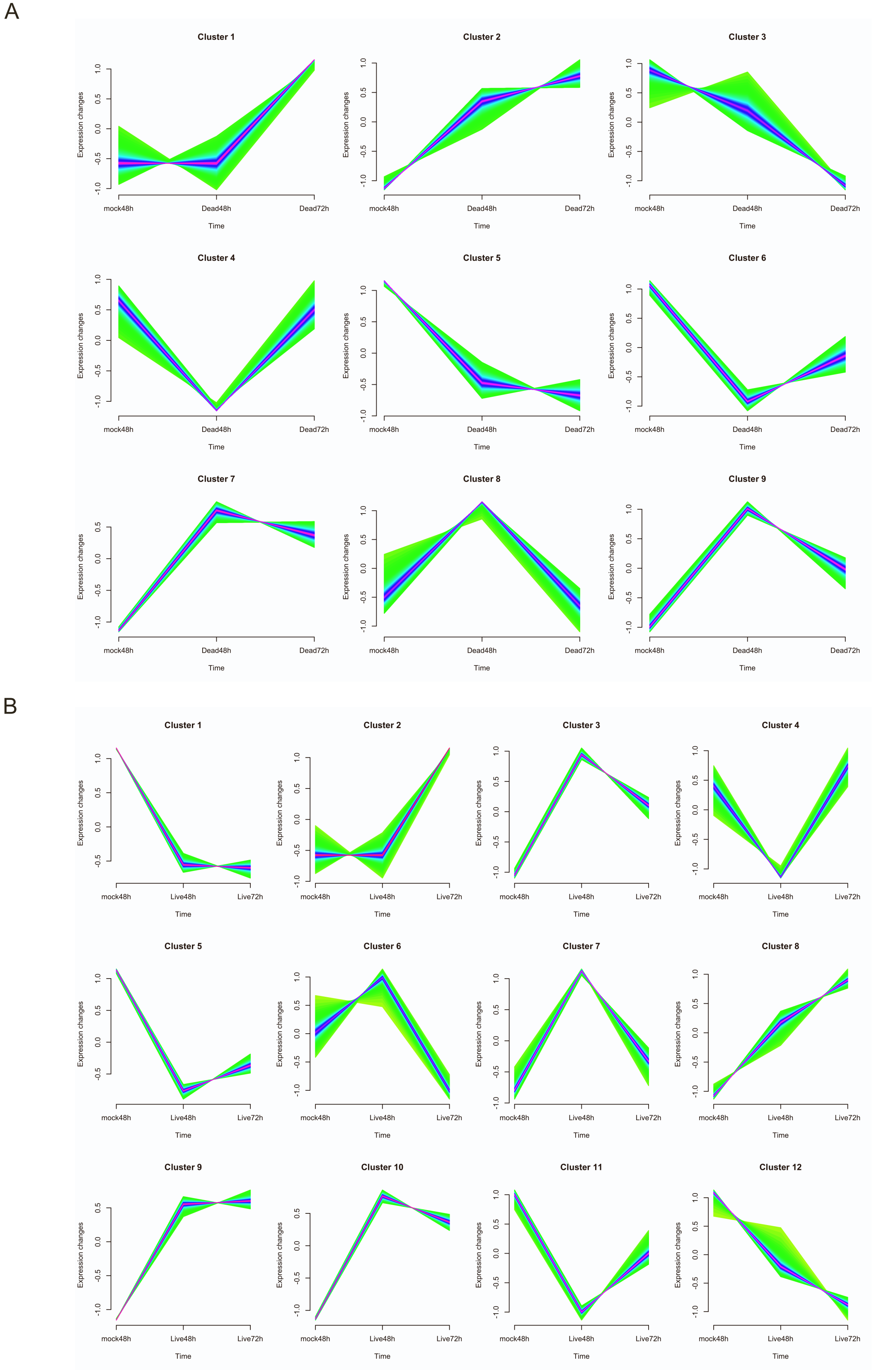

Figure S5. Longitudinal gene expression dynamics defined by Mfuzz clustering. Gene clusters were identified based on temporal changes across mock_48h, 48h, and 72h post-infection. A) Longitudinal profiles of 9 gene clusters in the DeadMtb group. B) Longitudinal profiles of 12 gene clusters in the LiveMtb group.

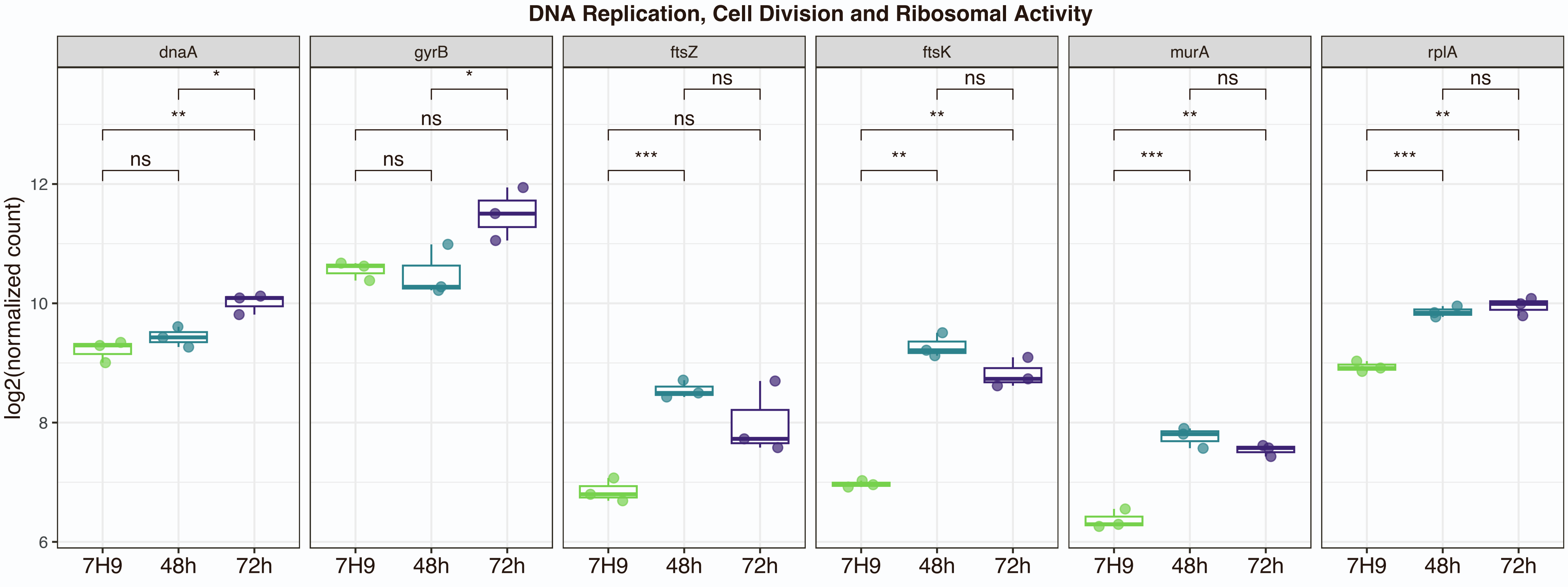

Figure S6. Expression of replication- and growth-associated genes in intracellular live Mtb versus 7H9 cultures. Shown are dnaA and gyrB (DNA replication), ftsZ and ftsK (cell division), murA (cell-wall synthesis), and rplA (ribosomal function). Each panel displays log_2 transformed normalized read counts for bacteria grown in 7H9 medium and for intracellular Mtb sampled at 48 h and 72 h (intra48h, intra72h). Statistical significance was assessed using the Wilcoxon test (*p < 0.05, **p < 0.01, ***p < 0.001).

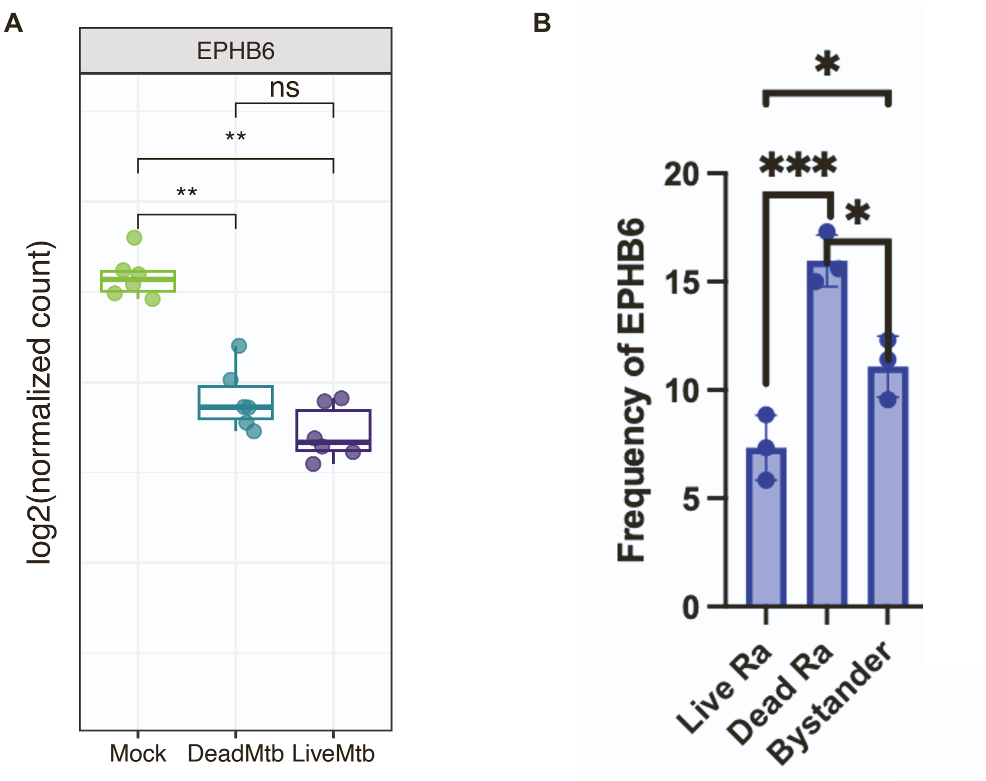

Figure S7. Expression of EPHB6 in macrophages upon H37Ra exposure. (A) Dual RNA-seq analysis showing EPHB6 expression in THP-1-derived macrophages under Mock, Dead, or Live Mtb conditions. Data from 48 h and 72 h post-infection were combined. (B) Flow cytometry analysis of EPHB6 expression in THP-1-derived macrophages 72 h post-infection, validating the RNA-seq results. Data are presented as mean ± SD from three independent experiments. Statistical significance was determined using one-way ANOVA（*$p < 0.05$, **$p < 0.01$, ***$p < 0.001$, ****$p < 0.0001$）.
